# TextNetTopics: Text Classification Based Word Grouping as Topics and Topics’ Scoring

**DOI:** 10.3389/fgene.2022.893378

**Published:** 2022-06-20

**Authors:** Malik Yousef, Daniel Voskergian

**Affiliations:** ^1^ Zefat Academic College, Zefat, Israel; ^2^ Computer Engineering Department, Al-Quds University, Jerusalem, Palestine

**Keywords:** text classification, topics detection, grouping, ranking, feature reduction, medical documents, latent dirichlet allocation (LDA), feature selection

## Abstract

Medical document classification is one of the active research problems and the most challenging within the text classification domain. Medical datasets often contain massive feature sets where many features are considered irrelevant, redundant, and add noise, thus, reducing the classification performance. Therefore, to obtain a better accuracy of a classification model, it is crucial to choose a set of features (terms) that best discriminate between the classes of medical documents. This study proposes TextNetTopics, a novel approach that applies feature selection by considering Bag-of-topics (BOT) rather than the traditional approach, Bag-of-words (BOW). Thus our approach performs topic selections rather than words selection. TextNetTopics is based on the generic approach entitled G-S-M (Grouping, Scoring, and Modeling), developed by Yousef and his colleagues and used mainly in biological data. The proposed approach suggests scoring topics to select the top topics for training the classifier. This study applied TextNetTopics to textual data to respond to the CAMDA challenge. TextNetTopics outperforms various feature selection approaches while highly performing when applying the model to the validation data provided by the CAMDA. Additionally, we have applied our algorithm to different textual datasets.

## 1 Introduction

The overwhelming amount of scientific research documents we are witnessing today leads to the importance of automatic text classification. The main aim of automatic text classification is to keep up with all the relevant published work by assigning each document to the appropriate predefined class according to its content (Uysal and Gunal, 2012).

One prevalent research problem within the text classification domain is medical document classification, where a medical document may comprise a medical record or a scientific research paper in the field. However, text classification in the medical domain is considered more challenging than that in other domains since it involves the manipulation of massive datasets, where the data is characterized by its high dimensionality and sparsity. We may find hundreds to thousands of medical characteristics in medical documents, such as complex terminologies, phrases, and abbreviations relevant to the medical field, which creates the issue of high dimensionality (Lee et al., 2017).

In this aspect, there are two main problems when dealing with high-dimensional datasets for text classification. First, processing a considerable amount of data increases to a large extent the computation time and entails more memory to run learning algorithms. In addition, it adds several parameters to the model and complicates it considerably. Second, some features are irrelevant, redundant, and add noise, degrading the classification performance (Liu and Motoda, 2008). Thus, selecting the most relevant feature subset is crucial so that the text classification process produces better results and ensures scalability.

The feature selection process or attribute reduction is the procedure of finding a subset of features that best represents by itself all the data. In the context of text classification, feature selection algorithms aim at representing a document with a reduced number of highly representative and discriminative features (Saeys et al., 2007).

Traditional feature selection algorithms in analyzing text are single feature ranking (Idris et al., 2015), a filter technique that chooses the top *m* features based on their rank as a subset from the total *n* features. Although it is a simple method with a low computation cost, it does not consider interactions between features. In fact, most filter methods evaluate features separately and cannot distinguish interactions between them (Garla et al., 2013).

Several studies conducted feature selection on biomedical text documents for text classification purposes. (Adriano Gonçalves et al., 2019) proposed a novel feature selection algorithm for full-text classification entitled “k-best-Discriminative-Terms”. For each class value, the average term frequency-inverse document frequency (TF-IDF) metric is calculated for each term; then, the difference is measured between corresponding values of terms in both classes to find frequent terms in one class but infrequent in the other. This study assessed the proposed method on Ohsumed corpora, which performed much better for full-text datasets than datasets containing a limited amount of text, such as title and abstract.

Abdollahi et al. (2019) proposed a novel two-stage approach. In the first stage, the most relevant concepts (features) were extracted from a domain-specific dictionary, namely, the Unified Medical Language System. In the second stage, particle swarm optimization is utilized to choose more related attributes from the extracted attributes in the first stage. The authors evaluated this approach on a widely used medical text dataset, called Integrating Biology and the Bedside (i2b2) dataset, and the results revealed a substantial improvement in the classification accuracy.

Parlak and Uysal (2018) explored the impact of feature selection methods on medical document classification utilizing MEDLINE corpora. The study compares two different feature selection approaches, namely the Gini Index and the Distinguish Feature Selector, using two pattern classifiers, the Bayesian network and the C4.5 decision tree. This study finds that combining the two proposed feature selection methods yields the best accuracy results.

The work proposed by (Sagar Imambi and Sudha, 2011) presents a new novel method for feature selection using the PubMed articles datasets. Their approach involved a preprocessing phase, where some Nature Language Process (NLP) takes were applied to the documents, e.g., tokenization, stemming, and stop words removal. Their method applies a variation of the Global Weighting Scheme, where the unique terms are extracted from documents and weighted through a global weighting schema proposed. The authors claim to achieve better results in terms of accuracy.

Apart from the mentioned studies, a recent direction suggests using the topic model notion as a feature reduction method, in which the text is represented as a mixture of hidden topics, where the extracted latent topics from text documents form the features (Onan et al., 2016). In other words, the textual data is represented as a bag of topics (Zhou et al., 2009; Yousef et al., 2020a) rather than a bag of words. However, in the short-text corpus, an advanced approach must be developed (Al Qundus et al., 2020).

Topic modeling is one popular technique in information retrieval that provides a convenient way to analyze themes from textual documents. Topic modeling techniques can discover unknown topics from a vast collection of documents and represent each extracted topic by word distributions with probabilities (Griffiths and Steyvers, 2004).

This paper proposes a TextNetTopic, a novel approach that applies feature selection by considering Bag-of-topics (BOT) rather than the traditional approach, Bag-of-words (BOW). Thus the approach performs topic selection rather than word selection. The rationale of the TextNetTopic is to score and rank the topics and use top *r* topics in the dataset that best discriminate the two classes of documents (in case we are dealing with a binary classification problem), where each topic contains a relatively small number of words (such as 10, 20, or 30).

The merit of the TextNetTopics tool is similar to some bioinformatics tools such as (Yousef et al., 2009; Yousef et al., 2019; Yousef et al., 2021a; Yousef et al., 2021b; Yousef et al., 2021c; Yousef et al., 2021d; Yousef et al., 2021e; Yousef et al., 2022) that rely on the model G-S-M (Grouping-> Scoring-> Modeling) developed by Yousef and his colleagues. For a review of such a model, refer to (Yousef et al., 2020b)**.**

In this study, we analyzed the performance of the TextNetTopic and three different widely-known feature selection methods: Extreme Gradient Boosting (XGBoost), Fast Correlation Based Filter (FCBF), and selectKBest (SKB), through four classifiers. These classifiers are Adaboost, Decision Tree (DT), Random Forest (RF), and LogitBoost. We analyzed the impact of feature selection on abstract-based biomedical document classification through a large dataset of PubMed papers (DILI relevant and non-relevant papers) compiled by CAMDA. In addition, we utilized a second dataset called the PubMed 20k RCT (Dernoncourt and Lee, 2017) for assessment, which was constructed upon the MEDLINE/PubMed baseline dataset for the sequential sentence classification task.

According to the empirical assessment performed in this study, the TextNetTopic tool can contribute to building a better text classifier with a small number of features and therefore extract more relevant papers from vast scientific papers repositories.

The rest of this paper is structured as follows: Section 2 explores the topic detection and the topics-based text representation. Section 2.1 presents our proposed approach in detail. Section 3 provides the evaluation of the TextNetTopic tool. Finally, Section 6 concludes the paper and presents future work.

## 2 Topic Detection

Topic Detection (also called Topic Modeling, Topic Analysis, or Topic Extraction) is a statistical technique with a group of algorithms for revealing, discovering, and annotating the hidden semantic structure from a huge volume of the document collection. The main aim of topic modeling is to discover patterns of word use, or themes, called topics, from a collection of unclassified text. Each discovered topic contains a cluster of words that frequently occur together and has a specific probability distribution over words. In addition, Topic Modeling connects documents that share similar patterns, assuming that each document has different proportions of topics. On the side of text mining, topic models rely on the bag-of-words assumption, which ignores the ordering of words (Alghamdi and Alfalgi, 2015) (Kherwa and Bansal, 2018).

In this aspect, four well-known topic modeling methods contribute to the text analysis in multiple domains, namely, Latent semantic analysis (LSA), Probabilistic Latent Semantic Analysis (PLSA), Latent Dirichlet Allocation (LDA), and Correlated Topic Model (CTM).

The latent semantic analysis is a method that utilizes statistical and mathematical computations applied to a large text corpus to extract and represent the contextual meaning and synonyms of words (Landauer et al., 1997). LSA uses singular value decomposition to reduce the high dimensionality of the vector space model (i.e., TF-IDF scheme). However, LSA can suffer from high mathematical complexity and has no strong statistical foundation (Hofmann, 1999). The probabilistic latent semantic analysis is another statistical method for analyzing data based on a latent class model. Compared to LSA, PLSA has a solid statistical foundation, and it can find latent topics and yield better performance (Hofmann, 1999).

On the other hand, the latent Dirichlet allocation is a generative probabilistic topic model that researchers widely utilize. The LDA is an unsupervised technique for topic discovery based on a hidden variable or latent topic model. The main idea of LDA is that each document in a corpus is represented as a random mixture of topics, and each topic represents a discrete probability distribution over a vocabulary that defines how likely each word is to appear in a given topic. Following this approach, each document is concisely represented by these topic probabilities (Alghamdi and Alfalgi, 2015). LDA is a parameterized method that involves several parameter values, i.e., the number of topics, the number of iterations, the alpha parameter for controlling the topic distribution per document, and the beta parameter for controlling the distribution of words per topic (Meir Blei, 2004).

### 2.1 Topics Used for Text Representations

Current research in the Text Classification field usually adopts Vector Space Model (VSM), also known as Term Vector Model, to represent documents. Using a VSM representation scheme, each document is encoded as a vector of identifiers (i.e., words; n-grams) with corresponding weights, such as Term Presence, Term Frequency, or TF.IDF.

However, this model suffers from the following limitations: 1) It has huge feature space, which results in high dimensional vectors. 2) The resulting vector is sparse (contains many 0’s). 3) It does not consider the exact ordering of words; each word is regarded as statistically independent. 4) It does not consider the grammar nor the semantic structure, and as a consequence, there is a semantic loss. 5) Two documents containing similar contexts but different vocabulary terms are not classified in the same category. Thus, a more compact representation scheme is needed to effectively avoid the mentioned problems (Eklund, 2018; Zrigui et al., 2012).

**FIGURE 1:**
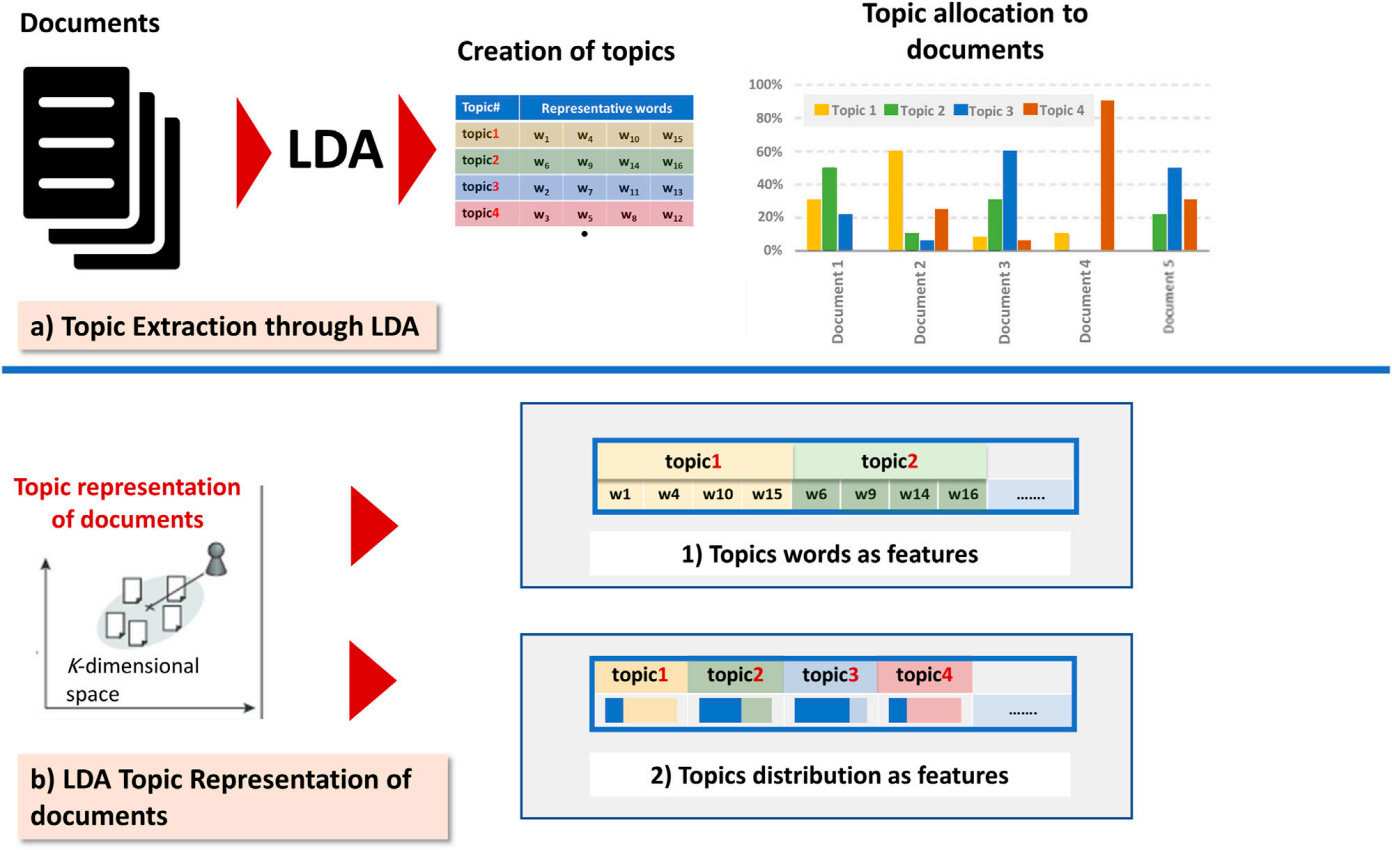
The two commonly utilized LDA representation schemes (topics words as features and topics distributions as features).

Recently, there has been progress in the document representation models; this progress is based on techniques that embed more and more syntactic and semantics of texts. Among various models, we quote the Latent Dirichlet Allocation (LDA); the basic idea is that a document is a mixture of hidden themes, called topics, and every topic is characterized by a probability distribution of words that are associated with it. Several studies used this generative model to represent text documents based on latent topics. Figure 1 illustrates the two commonly utilized LDA representation schemes (topics words as features and topics distributions as features). Since the number of topics is less than the number of terms in a collection, thus, topic-based representation schemes can reduce the spatial dimensions of a vector, consequently simplifying the computing processing and the running time.

Zrigui et al. (2012) have used LDA to index and represent the Arabic texts using the notion of topic encoding instead of the individual words. They extracted significant topics from the dataset, where a particular distribution of words describes each topic, then they represented each text document as a vector of these topics. In other words, each document is represented by a set of real-valued features, which comprise words contained in each topic. As a consequence, the dimensionality of the VSM vector is reduced while preserving more semantic information in the text representation.

Mo et al. (2015) have applied LDA for modeling topic distribution for each document in the dataset and trained a support vector machine classifier using distributions of topics as features. In other words, they have transformed documents into the form of a document-topic matrix, which contains the proportions of each topic in a document, and input such matrix into an SVM classifier. Their study compared the two feature representation schemes (LDA features and BOW features) and evaluated the impact of using a different number of topics in topic-based classification. Their results show that the topic distribution as features outperforms the traditional BOW features when applied to the task of automatic citation screening. (Wu et al., 1260) adopted the same representation scheme as in (Mo et al., 2015) for Chinese news documents and concluded from their experimental results that such a scheme produces better results in terms of classification accuracy and running time than the traditional TF-IDF method.

To this aspect, the majority of studies that utilized the LDA topic-based document representation (specifically topics words as features) used all extracted topics as the representative features, although it led to a significant dimensionality reduction of the text vector; however, some topics may be irrelevant and may add noise to the classification model. Thus our study will perform further feature reduction by conducting topic ranking to find *r* topics from the feature set (LDA extracted topics) that best discriminate the classes in the text classification process.

## 3 TextNetTopics

**FIGURE 2:**
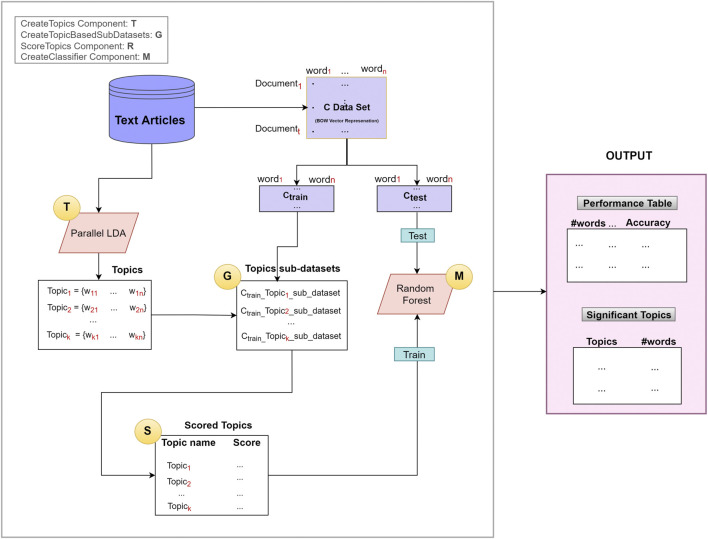
The TextNetTopics general approach is based on four main components: T for creating topics, G for generating topic-based sub-datasets, S for scoring/ranking topics, and M for creating and evaluating the model.

We have developed a novel approach called TextNetTopics, illustrated in Figure 2, based on G-S-M’s generic approach. The aim of the TextNetTopics is to score the topics (topic = group of words) and find the top significant *r* topics in the dataset that are used for training the classifier to best discriminate the two classes of documents (in case we are dealing with a binary classification problem), where each topic contains a relatively small number of words (such as 10, 20, or 30). The TextNetTopic tool can be applied to any domain in the text classification problem, and there is no domain restriction to the application of our proposed approach.

As illustrated in Figure 2, the TextNetTopics is consists of four main components (shown as circles):

1. T Component: for extracting topics using an LDA algorithm.
2. G Component: for generating topic-based sub-datasets.
3. S Component: for scoring and ranking topics.
4. M Component: for creating the classifier (Random Forest).

Let C represent the textual data set. We assume that we have *t* documents and *n* distinct terms (words) in our dataset C. The C data is split into C_train_ and C_test_. The C_train_ is used to score the topics and train the classifier to create the model, whereas C_test_ is used mainly to test and report the final performance. One might split it into 80% for training while the remaining 20% for testing.

### 3.1 Component T

**FIGURE 3:**
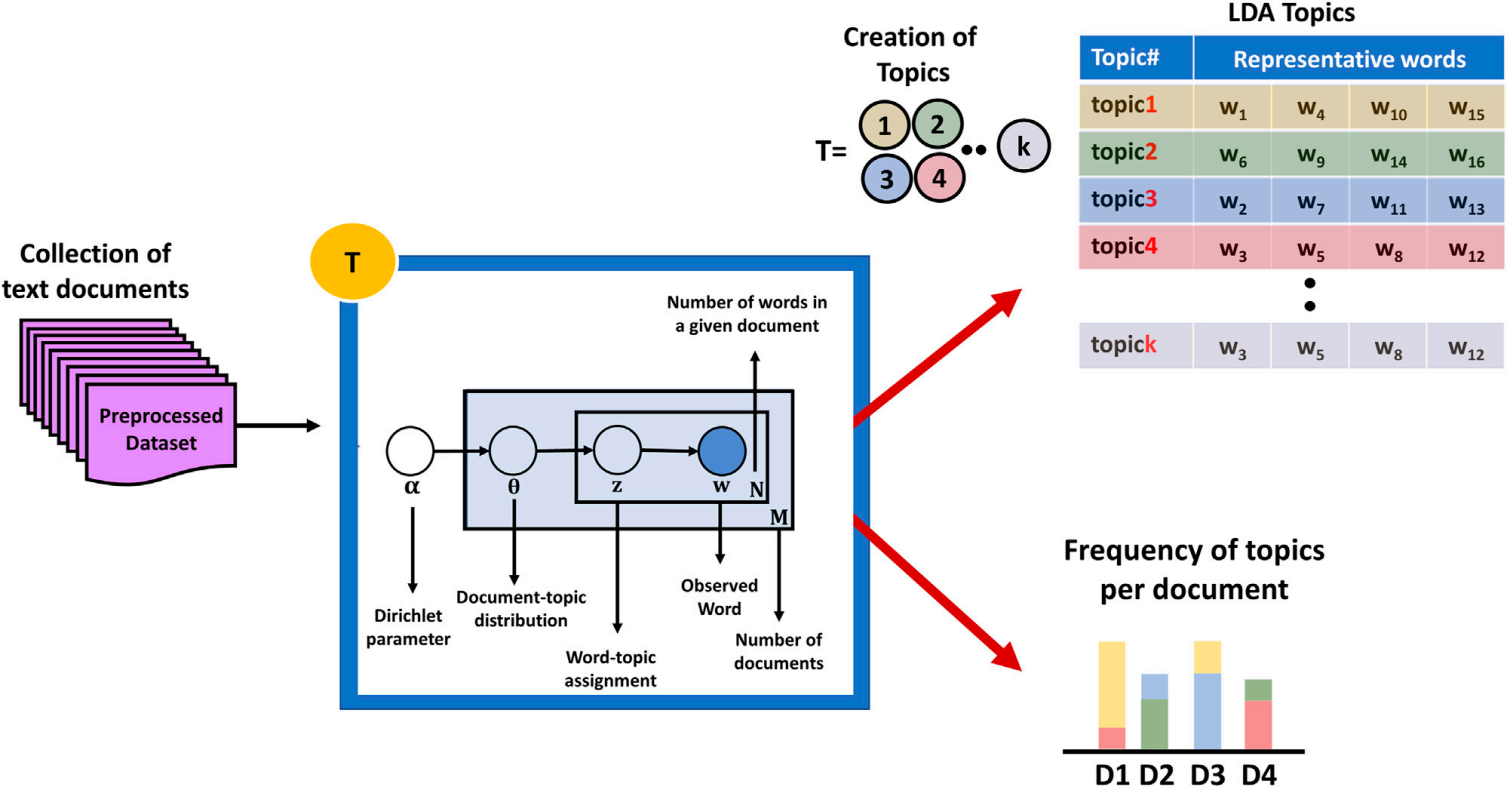
Component T working principle: extracting topics from a preprocessed dataset utilizing LDA.

The first component is the T component, which works on a preprocessed dataset (collection of documents) to detect the topics, where each topic is represented by a distribution probability of a set of words (refer to Figure 3). T component achieves this using a widely known topic modeling technique called LDA (David et al., 2003).

In this aspect, the LDA is a parameterized algorithm, where the user needs to set up some parameters, e.g., number of topics, number of words per topic, the alpha parameter, which defines the Dirichlet prior on the per-document topic distributions, and the beta parameter, which defines the prior on per-topic multinomial distribution over words. The output of the LDA algorithm is the detected *k* topics which is a group of words associated with their weight per topic, as illustrated in Table 1. Figure 3 provides detailed information on the T component.

**TABLE 1:** An example of topics detected by LDA applied on the CAMDA dataset.

Topic id	List of words
topic_0	“patient, disease, studi, risk, rate, percent, transplant, clinic, treatment, compar”
topic_1	“patient, treatment, therapi, studi, infect, clinic, receiv, week, safeti, efficaci”
topic_3	“patient, treatment, respons, cancer, studi, surviv, receiv, therapi, month, phase”
topic_4	“express”, “cell, hepat, infect, viru, hcv, activ, hbv, respons, protein, express”

Table 1 illustrates an example of topics detected by LDA, where each topic is represented by 10 words.

### 3.2 Component G

Component G is the grouping component. While the T component creates the topics that are the groups of words, the G component uses those topics to create the related sub-datasets.

Component G first generates sub-datasets derived from the original dataset (Bag-of-Word vector representation of the training dataset). Each sub-dataset corresponds to a specific topic from the previous step (LDA extracted topics) and consists mainly of terms/words belonging to that topic.

**FIGURE 4:**
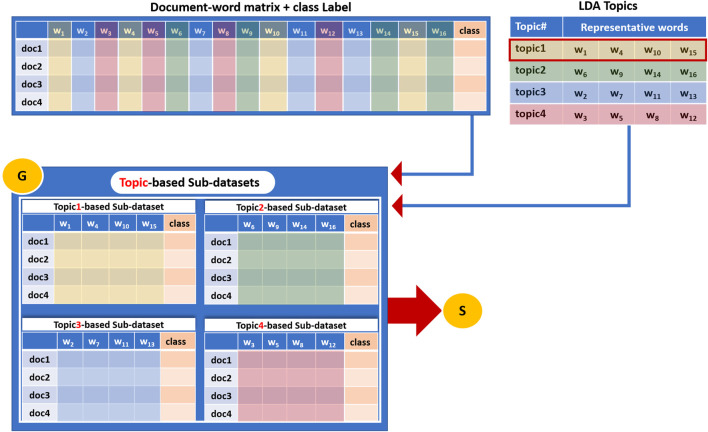
An example of how a sub-dataset is generated based on terms that belong to a topic and then subjected to the Scoring Component S.

In this aspect, each sub-data set contains the relative term frequency values for only the terms included in the topic and is associated with the original class labels (positive, negative). Figure 4 illustrates how a sub-dataset is generated based on terms that belong to a topic. This study will refer to each sub-dataset as Topic_i__sub_dataset, where i = 1, 2 ... , k, where k = size (T) corresponds to the number of topics extracted by the LDA algorithm.

Figure 4 illustrates an example of a dataset that consists of four documents and fifteen distinct terms/words. The class column represents the label of each document that might be a positive or negative label. The G component takes as input the LDA topics (Figure 4 upper right table) and the document-word representative matrix (Figure 4 upper left table) and creates four sub-datasets as an output, each corresponding to one topic. These sub-datasets will serve as input to the S component to perform the scoring step. Thus, four sub-datasets (corresponds to the number of topics k = 4) are created, and each one contains features/terms/words belonging to one topic.

### 3.3 Component S

**FIGURE 5:**
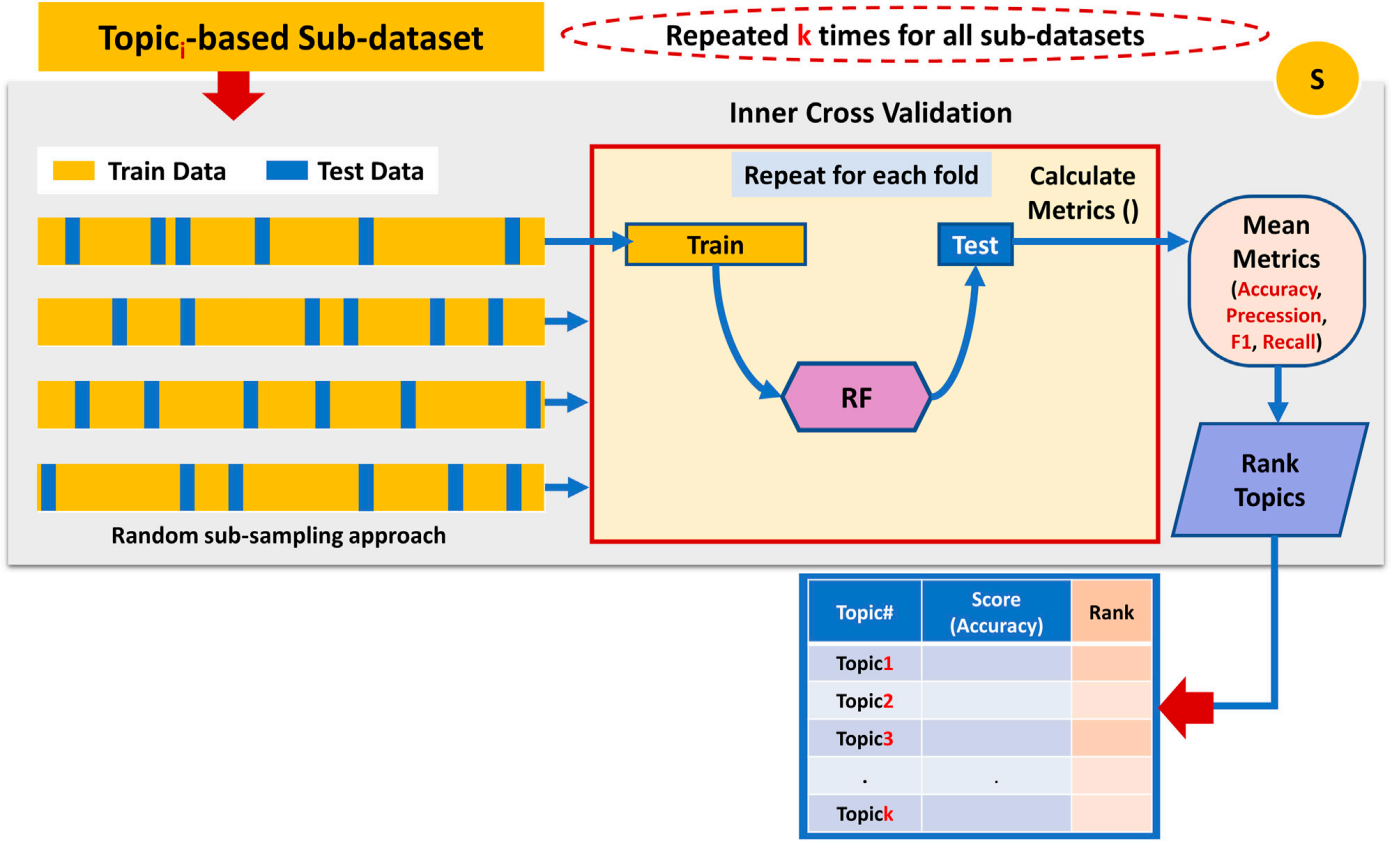
Performing internal cross-validation on the topic-based sub-dataset to assign a score to the associated topic.

The third component is the *S* component, which takes as input all the topic-based sub-datasets generated by Component G, as seen in Figure 5. This component utilizes a machine learning algorithm (Random Forest) with an internal Monte-Carlo stratified cross-validation (MCCV) applied on each sub_dataset to assign a score for each topic. Stratified sampling ensures that the class labels are equally distributed within the training and testing data. The scoring is measured by testing the ability of each topic to predict the class label. In other words, the score is a prediction value of how well the documents can be classified based on those terms that belong to a topic. Among the various standard performance metrics measured (i.e., Accuracy, Precession, Recall, F1 score), this component uses the mean classification accuracy as the major performance metric to score the topic.

Once all topics get a score, component S ranks them accordingly. For example, the topic with the highest mean accuracy performance gets the top rank.

### 3.4 Component M

The last component is responsible for training and creating the model, which considers topics rather than words when applying feature selection. This component builds a Random Forest model for the top-ranked topics in an accumulated order and reports the cumulative performance of the model. In each iteration, component M generates a new sub-data corresponding to a set of topics ready for training and testing. In this aspect, since different topics may contain duplicate words, accumulated topics will consist only of distinct words.

**FIGURE 6:**
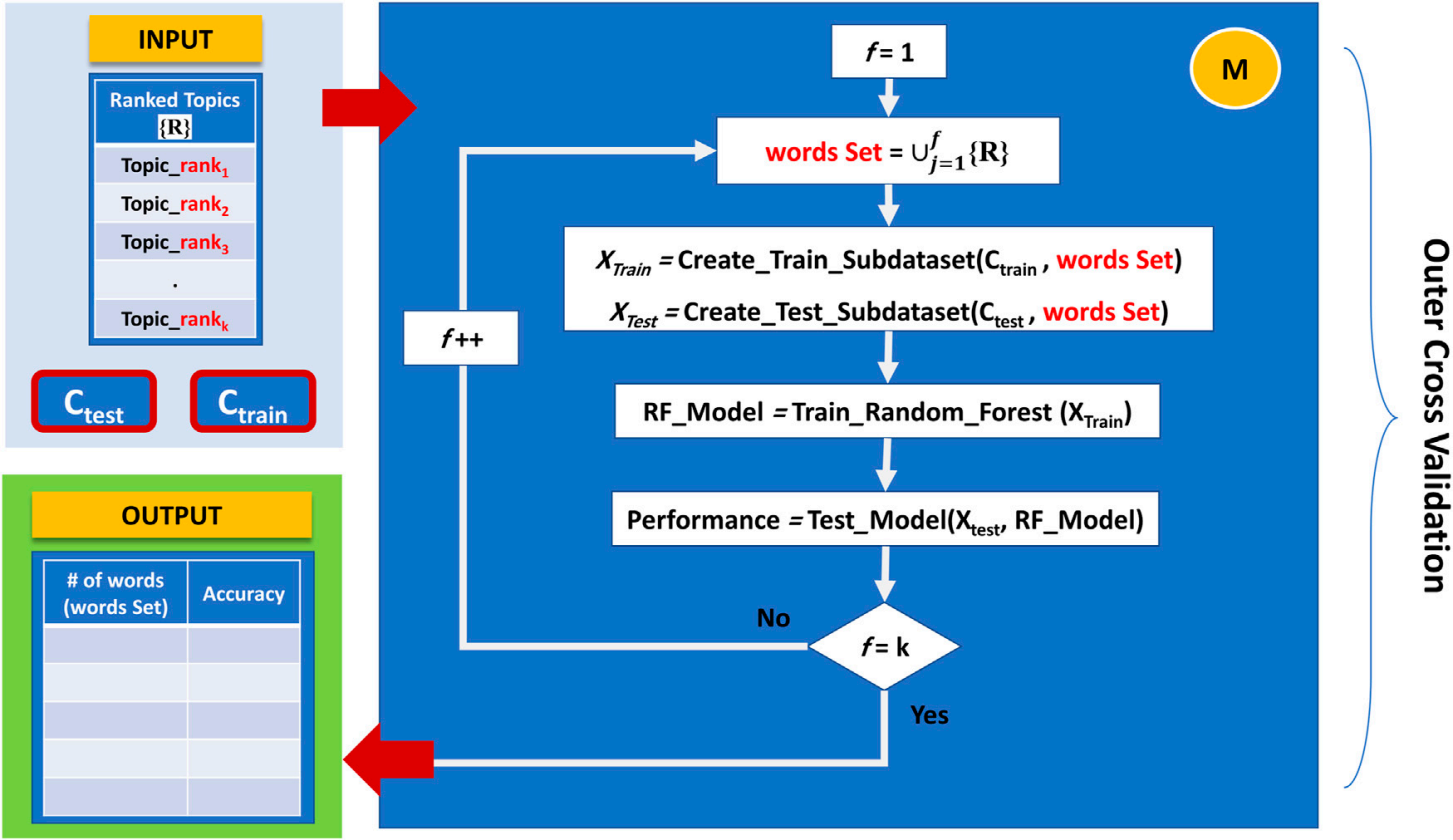
The working principle of Component M: finding the best feature set, in terms of topics terms combinations, that provides the best performance.

Initially, component M starts by building a Random forest model utilizing only the terms that belong to the top-ranked topic. The second iteration takes the top-ranked topic terms set and accumulates it with the terms sets that belong to the second-ranked topic to create a new sub-data; this sub-data will be directed for training and testing the model, and so on. This method will continue until we process all topics in the same way. Figure 6 demonstrates the working principle of Component M.

By following this approach, we can plot the performance results over different constructed feature sets (i.e., the top 1 ranked topic, top 2 ranked topics, till top 10 ranked topics) and find the best feature set in terms of topics terms combinations that provides the best performance.

## 4 Experimental Work

The following subsections briefly describe the utilized dataset, the methods used to process the dataset, the success measures, and finally, the experimental results.

### 4.1 Dataset

The empirical evaluation in this study was carried out using the Drug-Induced Liver Injury (DILI) dataset provided by CAMDA. CAMDA compiled a large set of PubMed papers relevant to DILI to form the positive class and another set of unrelated papers to form the negative class. In this dataset, the title and the abstract section are collected only.

The positive data comprises about 14,000 DILI-related papers referenced in the NIH LiverTox database (Adriano Gonçalves et al., 2019), which have been validated by a panel of DILI experts. This positive reference is split 50:50 into one part released for the challenge and one withheld for final performance testing. This is complemented by a realistic, non-trivial negative reference set of about 14,000 papers that is highly enriched in manuscripts that are not relevant to DILI but where obvious negatives and any positives we could identify have been removed by filtering for keywords and through well-established language models, followed by a selective manual review by DILI experts at the FDA. This negative reference is also split 50:50 into one part released for the challenge and one withheld for final performance testing.

The data utilized in the training process consisted of 7097 positive papers and 7026 negative papers. However, we observed that 2016 of the positive papers contained an empty abstract, as opposed to the negative papers, with no empty abstracts.

CAMDA provided two validation datasets. The first validation dataset contains 14211 papers without any information regarding their class labels and includes 1982 papers without abstracts. The second validation dataset contains highly unbalanced data of 2000 unlabeled papers. The negative paper set has been expanded considerably to reflect the low prevalence of DILI-relevant papers; this will provide an additional assessment of how well the model works when applied in this scenario.

Finally, the output of TextNetTopics on these datasets was submitted to CAMDA for evaluation. The returned results are reported in the result section. Table 2 presents the characteristics of the utilized DILI dataset.

**TABLE 2:** Distribution of classes in the dataset samples.

	The number of relevant papers	The number of non-relevant papers
Full Dataset	∼14,000	∼14,000
Training DS	7097	7026
Validation DS#1	14211 papers (labels are withheld part for final performance testing)	
Vlidation DS#2	2000 papers (labels are withheld part for final performance testing)	

In addition, we have utilized a second dataset called the PubMed 20k RCT (Dernoncourt and Lee, 2017), which is constructed upon the MEDLINE/PubMed baseline dataset for the sequential sentence classification task. The dataset consists of approximately 20,000 medical abstracts, particularly in randomized controlled trials (RCTs), with 1 million sentences. The dataset is split randomly into three sets: a training set of 15000 abstracts and testing and validation sets with 2500 abstracts for each. In this aspect, each record corresponds to either PMID or a sentence with its label, where each sentence is annotated with its role in the abstract using one of the following five classes: background, objective, method, result, or conclusion.

This study transformed the dataset from a multi-class dataset into a binary dataset to evaluate the TextNetopic tool. Moreover, to ensure a near-balanced dataset, we considered the largest class as a positive class and the remaining classes as negative classes. We considered the ‘Method’ category as the positive class in this context.

### 4.2 Text Pre-Processing

Since the raw form of documents within the dataset contains a lot of noisy data, a text-preprocessing step is necessary to get better analysis and reduce the dimensionality of input data since many words are useless and their existence does not have any impact on the text classification. The text-preprocessing involved the following NLP tasks utilizing knime (Berthold et al., 2009) workflows:

• Removing all punctuation characters.
• Filtering out all words with less than three characters.
• Striping all terms that consist mainly of digits (Number filtering).
• Converting all words into lowercase (Case-folding).
• Removing stop words utilizing the built-in English stop word list within the Stop Word Filter node.
• Stemming all words utilizing the snowball stemming library.
• Filtering out all terms with minimum document frequency (terms that appear in less than 1% of the total number of documents in the corpus are removed).
• Keeping English texts only using a language detector provided by Tika-collection.

In this aspect, the number of words/features after performing the pre-processing stage for the CAMDA dataset is 1158 words.

### 4.3 Text Vectorization (Feature Extraction)

To feed the dataset to the TextNetTopic tool or any machine learning model, we need to produce a structured form of data. The vectorization process converts unstructured free text to a structured numerical format called a feature vector. One commonly used is the Bag-of-words (BOW) model. In our project, we created a BOW feature vector representation for each document in the dataset. Each vector is represented in the terms space, where its dimensions are specified by the number of distinct terms of the preprocessed dataset (BOW). The BOW can be represented by Term-Frequency (TF) or binary. The TF format counts the number of times a word appears in the document, while the binary representation only distinguishes between 1 if the word is present and 0 otherwise. In our case, we will use the relative TF format, where the values of a document vector are calculated by taking the ratio of the term count to the document size (total number of words in a document).

### 4.4 Evaluating TextNetTopic Performance

To assess TextNetTopc empirically, we have compared the performance of the TextNetTopic tool with three different widely-known feature selection methods through four classifiers, namely Adaboost, Decision Tree (DT), Random Forest (RF), and LogitBoost.

The three feature selection methods utilized in this study are:

• Fast Correlation Based Filter (FCBF) (Senliol et al., 2008) is a multivariate feature selection method that starts with a complete set of features, uses symmetrical uncertainty to calculate the dependencies of features, and finds the best subset using the backward selection technique with the sequential search strategy. It is a correlation-based feature subset selection method that runs, in general, significantly faster than other subset selection methods.
• Extreme Gradient Boosting (XGBoost) (Chen and Guestrin, 2016) is used for supervised learning problems. XGBoost is a decision-tree-based ensemble Machine Learning algorithm that uses a gradient boosting framework. A benefit of using gradient boosting is that after the boosted trees are constructed, it is relatively straightforward to retrieve importance scores for each attribute. Generally, importance provides a score indicating how useful or valuable each feature was in the construction of the boosted decision trees within the model. The more an attribute is used to make key decisions with decision trees, the higher its relative importance.
• selectKBest (SKB): SKB scores the features against the class label using a function and selecting features according to the k highest score.

The metric used for the assessment of the classifier performance isthe F1-measure, which is a harmonic mean of precision and recall.

### 4.5 Experimental Setup

We have implemented the TextNetTopics tool in Knime platform, where we have considered the workflow used in (Wu et al., 1260). All experiments in this study were executed on the same workstation (computing environment).

To estimate the TextNetTopics performance, we used a stratified Monte Carlo Cross-Validation (MCCV) as the evaluation method. MCCV splits the dataset randomly into two sets; 90% of records as a training set and the remaining 10% as a testing set. We repeated this process one hundred times to ensure that most records appeared in both sets (train/test) and explore more possible partitions in the evaluation than its counterpart, the k-fold cross-validation. This study has chosen stratified sampling since it preserves the same distribution of samples for each class in each split; thus, we can ensure that the obtained performance results are less biased or less optimistic than the simple hold-out method.

Concerning the component G in TextNetTopic that is responsible for detecting topics out of a collection of documents, it utilized the LDA implementation in Knime, which is a simple parallel threaded implementation of LDA, following Newman, Asuncion, Smyth, and Welling, Distributed Algorithms for Topic Models (Parlak and Uysal, 2018). This study considers two main LDA parameters. The number of topics (n_topics) and the number of words per topic (n_topics_word). We have chosen n_topics = 20 and n_topics_words = 20 because they yield slightly better performance results than other values. However, one might need to run a greedy search to find the optimal values. The alpha parameter that defines the Dirichlet prior on the per-document topic distributions and the beta parameter that defines the prior on per-topic multinomial distribution over words are set to their default value, 0.1 for each.

We have used the Random Forest as the main machine learning algorithm for scoring topics and training the classifier. Finally, XGBOOST, FCBF, and SKB feature selection methods are applied using the skfeature and sklearn libraries in Python 3 (David et al., 2003).

## 5 Results

Table 3 provides various performance metrics of the TextNetTopics for different values of n_topics (number of topics) and n_topics_words (number of words per topic) for the CAMDA dataset. We have chosen n_topics = 20 and n_topics_words = 20 because they yield slightly better performance results than other values.

**TABLE 3:** TextNetTopics performance metrics for different values of n_topics and n_topics_words for the CAMDA dataset over ten iterations. The top ∼70 features are considered for comparison.

#Topics	#Words	#Terms (mean)	Accuracy	Sensitivity	Specificity	F1 score	AUC	Precision
10	10	67	0.914	0.905	0.923	0.913	0.969	0.922
20	10	70	0.917	0.911	0.922	0.917	0.972	0.922
40	10	65	0.921	0.917	0.925	0.921	0.971	0.925
60	10	64.5	0.919	0.918	0.919	0.919	0.967	0.920
10	20	69.0	0.921	0.915	0.926	0.920	0.974	0.926
20	20	75.2	0.924	0.920	0.927	0.924	0.970	0.928
40	20	73.2	0.917	0.913	0.921	0.917	0.968	0.921
60	20	73.0	0.917	0.920	0.914	0.917	0.966	0.915
10	30	75.3	0.920	0.917	0.924	0.920	0.973	0.924
20	30	76.5	0.920	0.915	0.926	0.920	0.972	0.926

Table 4 presents the performance results of TextNetTopics over different constructed feature sets (i.e., the top 1 ranked topic, top 2 ranked topics, till top 10 ranked topics) of the CAMDA training data.

**TABLE 4:** TextNetTopics performance over top topics for CAMDA dataset. #Accumulated_Topics column is the number of significant topics, #Words is the average of words on each level over the 100 iterations.

#Accumulated_Topics	#Words	Accuracy	Sensitivity	Specificity	F1 score	AUC	Precision
11	104.14	0.93	0.92	0.93	0.93	0.98	0.93
10	94	0.93	0.92	0.93	0.93	0.98	0.93
8	75.3	0.92	0.92	0.92	0.92	0.97	0.93
6	62	0.92	0.92	0.92	0.92	0.97	0.92
4	49	0.91	0.91	0.91	0.91	0.96	0.91
2	31.94	0.89	0.90	0.89	0.89	0.95	0.89
1	20	0.87	0.88	0.87	0.88	0.93	0.87

After the model was created, it was applied to two validation data provided by CAMDA. The results were sent to CAMDA for evaluation. The received results are presented in Table 5 in the first two rows, namely, “Text Topics/V1” for the first validation data and “Text Topics/V2” for the second validation data. The F1 scores were 0.92 and 0.88 for the first and second validation data, respectively.

**TABLE 5:** TextNetTopics performance results over two validation datasets provided by CAMDA.

	Accuracy/stdv	Recall	Precision	F1 score
TextNetTopics/V1	0.92	0.92	0.92	0.92
TextNetTopics/V2	0.87	0.94	0.82	0.88

To this end, we have compared the performance of the TextNetTopic tool and three different feature selection methods, namely Extreme Gradient Boosting (XGBoost), Fast Correlation Based Filter (FCBF), and selectKBest (SKB), through four classifiers. These classifiers are Adaboost, DT, RF, and LogitBoost. Table 6 presents the results, where the highest F1 scores are highlighted in bold text.

**TABLE 6:** Results for different algorithms with different feature selections applied to the CAMDA dataset. The top 100 features are considered for each feature selection algorithm. The standard deviation of the accuracy is present in the Accuracy column after the “slash” sign.

	FS	Accuracy/stdv	Recall	Precision	F1 score
TextNetTopics		**0.93/0.006**	0.93	**0.93**	**0.93**
Adaboost	XGBOOST	0.79/0.05	0.79	0.82	0.79
DT	XGBOOST	0.76/0.05	0.78	0.78	0.76
LogitBoost	XGBOOST	0.79/0.04	0.79	0.82	0.79
RF	XGBOOST	0.77/0.05	0.81	0.78	0.78
Adaboost	SKB	0.91/0.007	0.90	0.92	0.91
DT	SKB	0.88/0.01	0.87	0.88	0.88
LogitBoost	SKB	0.91/0.008	0.91	0.91	0.91
RF	SKB	**0.93/0.007**	0.93	0.92	**0.93**
Adaboost	FCB	0.70/0.03	0.89	0.65	0.75
DT	FCB	0.52/0.05	**0.97**	0.52	0.67
LogitBoost	FCB	0.71/0.03	0.89	0.65	0.75
RF	FCB	0.57/0.08	0.90	0.56	0.68

Bold values represent the highest values in each metric column.

We can notice from those results that our novel method “TextNetTopics” achieved in most cases a higher F1 score than other feature selection methods combined with different machine learning algorithms, except the result from the RF classifier when combined with SKB feature selection method, which has similar F1-score.

On the other hand, Table 7 presents the performance results of TextNetTopics over different constructed feature sets (i.e., the top 1 ranked topic, top 2 ranked topics, till top 10 ranked topics) of the PubMed 20k RCT training data.

**TABLE 7:** TextNetTopics performance over top topics for PubMed 20k RCT dataset. #Accumulated_Topics column is the number of significant topics, #Words is the average of words on each level over the 100 iterations.

#Accumulated_Topics	#Words	Accuracy	Sensitivity	Specificity	F1 score	AUC	Precision
10	74.96	0.83	0.74	0.90	0.81	0.88	0.84
8	65.54	0.83	0.74	0.90	0.80	0.88	0.84
6	54.26	0.82	0.71	0.90	0.76	0.87	0.83
4	41.74	0.81	0.69	0.90	0.75	0.86	0.82
2	27.54	0.80	0.66	0.90	0.73	0.84	0.82
1	17.94	0.79	0.63	0.90	0.71	0.82	0.82

A comparison of the performance of TextNetTopics against the performance of other feature selection techniques for PubMed 20k RCT dataset is presented in Table 8. Due to a significant imbalance between relevant and irrelevant instances in the utilized dataset, accuracy will not be a good indicator of an effective performance; thus we place a particular emphasis on F1-score. According to the results, XGBoost feature selection technique with LogitBoost classifier outperformed other methods, including TextNetTopics, and achieved higher F1-score, AUC, precision, specificity, and accuracy, except for recall. Although TextNetTopic did not get the highest results, the performance metrics obtained are comparable with the other methods with a 0.80 F1-score.

**TABLE 8:** Results for different algorithms with different feature selections applied on PubMed 20k RCT dataset. The top 60 features are considered for each feature selection algorithm.

	FS	Accuracy	Recall	Specificity	F1 score	AUC	Precision
TextNetTopics		0.83	0.74	**0.90**	0.84	0.88	0.80
Adaboost	XGBOOST	0.86	0.82	0.89	**0.85**	0.92	0.83
DT	XGBOOST	0.80	0.73	0.84	0.76	0.79	0.74
LogitBoost	XGBOOST	**0.87**	0.83	**0.90**	**0.85**	**0.93**	**0.84**
RF	XGBOOST	0.85	0.81	0.88	0.83	0.91	0.82
Adaboost	SKB	0.86	0.81	0.89	0.84	0.92	0.83
DT	SKB	0.79	0.72	0.84	0.76	0.79	0.74
LogitBoost	SKB	0.86	0.83	0.89	0.84	**0.93**	0.83
RF	SKB	0.85	0.82	0.88	0.82	0.91	0.82
Adaboost	FCB	0.62	0.84	0.47	0.53	0.66	0.65
DT	FCB	0.49	**0.89**	0.22	0.46	0.60	0.59
LogitBoost	FCB	0.62	0.85	0.46	0.53	0.66	0.65
RF	FCB	0.57	0.83	0.39	0.50	0.65	0.62

One might justify the obtained results for TextNetTopics due to the fact that topics are scored based on considering all their representative words at a time, and this might influence the performance, while the other feature selection approaches score each word individually and rank them accordingly, they don’t consider a group of words together as our tools perform.

## 6 Discussion and Conclusion

In this paper, we have proposed a novel text classification approach called TextNetTopics, that selects topics as features rather than individual words as features for training the classifier, hence performing dimensionality reduction while preserving the semantic descriptions of text documents. Our approach is based on four main components. The T Component is for extracting topics using an LDA algorithm. The G Component generates topic-based sub-datasets. The S Component performs the scoring and ranking topics, and finally, the M Component creates the classification model. Utilizing this architecture, we can reduce the dimensionality of the vector space model while embedding more semantic information into the text document representations. After performing an in-depth investigation to measure the performance of TextNetTopic and its effectiveness, TextNetTopics was able to achieve high performance in most cases compared to other feature selection algorithms due to its ability to utilize the semantic structure of the text documents rather than just considering frequencies of words. The novelty of the TextNetTopics approach lies in the fact that it scores topics considering all its representative words, which might be considered as one of the limitations of the tool that influence the performance; for example, some members (words) within topics may have a noisy impact and hinder the classification performance. The other feature selection approaches, on the other hand, consider each word individually and are not restricted to considering a group of words together as our tool performs. Future work will study how one can also score the members of each topic and suggest considering those with high scores for training the model. An additional limitation of TextNetTopics is caused by using LDA that requires setting the k number of topics and must be known ahead of time. Future work will be to detect the optimal number k of topics. In the current version of TextNetTopics, we have performed some preprocessing steps to detect the optimal number of k that might improve the performance.

In the area of feature selection or feature ranking (scoring), one of the questions that may arise is: is it possible that two features that are useless when considered separately can be useful together (combined)? The answer is yes. In TextNetTopics, the scoring component treats each topic individually, while one future approach would develop the S component to score topics simultaneously.

## Data Availability

The TextNetTopic knime workflow can be download from: https://kni.me/s/GrzIa-oh0Mv9Qm17 Or https://github.com/malikyousef/TextNetTopics.git.
